# Micro- and Nano-Scales Three-Dimensional Characterisation of Softwood

**DOI:** 10.3390/jimaging7120263

**Published:** 2021-12-03

**Authors:** Alessandra Patera, Anne Bonnin, Rajmund Mokso

**Affiliations:** 1 Section of Turin, National Institute for Nuclear Physics, 10125 Turin, Italy; 2 X-ray Tomography Group, Swiss Light Source, Paul Scherrer Institute, 5232 Villigen, Switzerland; anne.bonnin@psi.ch (A.B.); rajmund.mokso@psi.ch (R.M.); 3 Department of Physics, Technical University Denmark, 2800 Kgs. Lyngby, Denmark

**Keywords:** non-rigid strain, phase-contrast X ray imaging, softwood, swelling/shrinkage

## Abstract

Understanding the mechanical response of cellular biological materials to environmental stimuli is of fundamental importance from an engineering perspective in composites. To provide a deep understanding of their behaviour, an exhaustive analytical and experimental protocol is required. Attention is focused on softwood but the approach can be applied to a range of cellular materials. This work presents a new non-invasive multi-scale approach for the investigation of the hygro-mechanical behaviour of softwood. At the TOMCAT beamline of the Paul Scherrer Institute, in Switzerland, the swelling behaviour of softwood was probed at the cellular and sub-cellular scales by means of 3D high-resolution phase-contrast X-ray imaging. At the cellular scale, new findings in the anisotropic and reversible swelling behaviour of softwood and in the origin of swelling hysteresis of porous materials are explained from a mechanical perspective. However, the mechanical and moisture properties of wood highly depend on sub-cellular features of the wood cell wall, such as bordered pits, yielding local deformations during a full hygroscopic loading protocol.

## 1. Introduction

Cellular structures, made of different materials and in different geometries, are found widely in nature and inspire the development of many artificial man-made materials. Examples of cellular structures in nature are wood, coral, plant steams, glass sponges, cork and cancellous bone. These materials are made of an interconnected network of beams and/or plates resulting in a much lower density than the bulk material. The physical, mechanical and thermal properties such as density, conductivity, stiffness and strength of the bulk material can be dramatically changed by several orders of magnitude when organised into a cellular structure [1]. Understanding the mechanical response of cellular materials subjected to environmental stimuli is crucial for optimum material design. The complex swelling behaviour of natural cellular materials with different architecture (e.g., geometrical heterogeneity, multi-layered cell wall) needs further investigations and the role of the geometry of cellular materials and cell wall composition on their hygro-mechanical properties should be assessed. Such studies must be performed at cellular and subcellular levels to capture the exact mechanism at play. This paper describes the moisture induced swelling, with respect to the dimensional stability of spruce wood. Two phenomena are considered of interest: shape memory and buckling effect. Moisture activated shape memory may be of interest in biomedical applications. The moisture shape memory effect is seen in a variety of polymers and biological materials [2,3]. Further investigations based on experiments and combined with a modelling approach are required for understanding the mechanisms that govern the ability of certain materials to recover their original shape after moisture loadings. On the other hand, moisture effects such as buckling may cause undesired internal effects, including local deformations and even cracking due to an internal restraint. For these reasons, wood is an interesting material for setting up a robust multi-scale experimental approach to study the deformations at the micro- and nano-scales. Such an approach can also be used for the study of other biological and cellular materials.

**Figure 1:**
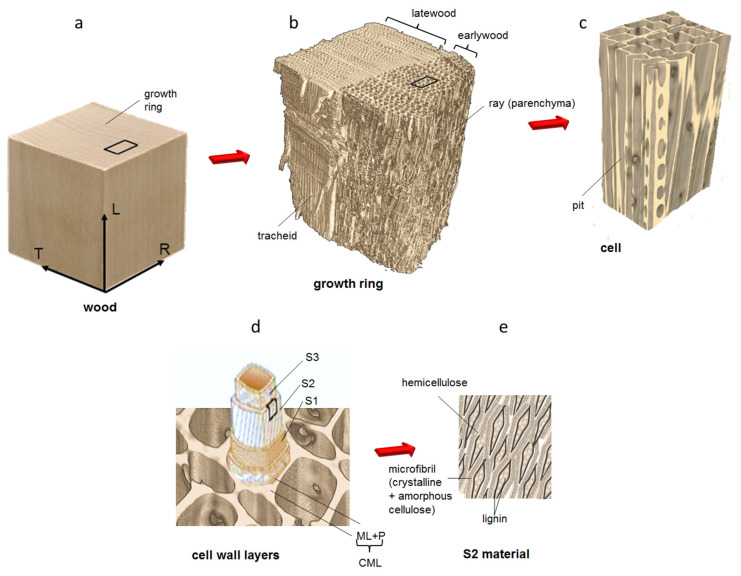
Schematic representation of the hierarchical structure of wood: from macro to micro scales, showing: (**a**) the growth ring, (**b**) the latewood and the earlywood tissues, (**c**) singularities in the cell wall, such as pits, (**d**) the cell wall layers and (**e**) the organisation of the chemical components.

Softwood is an orthotropic material composed of two types of cells, named tracheids (85–95%) and ray cells (5–12%), as can be seen in Figure 1. The inner void volume of the cell is called the cell lumen and the material surrounding the lumen is named the cell wall. In temperate regions with seasonal climatic cycles, softwood tissues vary over the growing season. During spring, the tissue, called earlywood, has thin cell walls and large lumens. As the growth season progresses, the cells become smaller and the cell walls thicker. This part is named latewood and it is denser than earlywood. The region between earlywood and latewood may lead to transition wood. The bordered pit represents an important feature in softwood as it controls the transport of water between cells. Bordered pits are passages through the cell walls of xylem conduits (vessels and tracheids) formed by a separation between the secondary walls from the compound middle lamella. The entry opening has a diameter of 3–7 μm and is called the porus or pit aperture. In the central plane, a membrane spans the pit chamber, with its thick centre called the torus (diameter 5–10 μm, thickness 100 to 500 nm depending on the species). The observation of the mechanical properties of wood with different mechanical forces has been impressed upon by many researchers over the last century at the macro scale (lumber) and, recently, at the cellular scale with microscopy, such as that reported in [4,5,6,7], or with X-ray tomographic microscopy, as reported in previous works by authors in [8,9,10]. The macroscopic swelling and shrinkage strains for spruce wood samples occur between dry state and 28% moisture content (MC, in ratio of mass of water to mass of dry wood). In the hygroscopic range, the strains are typically in the order of 0.5% longitudinally, 4% radially and 6% along the tangential direction. Strain hysteresis between swelling and shrinkage as a function of moisture content has been observed at the macroscopic scale to be in the range of 1% for maple. The cellular and the growth ring structures both affect the resulting moisture-induced deformations at the macroscale, as mentioned by [11]. Previous studies have found that μCt could be used to determine differences between earlywood and latewood shrinking and swelling in Picea abies wood samples [9,10]. More recently, a combination of X-ray scattering, neutron scattering and spectroscopic data was used to explore how the constituent polymers of wood cell wall interact to resist tension [12]. Dimensional change occurs due to a change in MC. Water molecules entering the cell walls result in an increase in dimensions of the cell wall material (swelling). Similarly, water molecules are removed from the cell walls upon drying the wood, causing the dimensions to decrease (shrinkage). X-ray tomography has been used to characterize swelling/shrinkage behaviour in the hygroscopic moisture range of wood tissue [13,14,15] and on micro-pillars cut out of cell wall material [16]. In this work, a multi-scale approach is presented to document the hygro-mechanical properties of spruce softwood (Picea abies) at cellular and sub-cellular scales. In addition, unprecedented achievements about the 3D behaviour of bordered pits in the wood cell wall during a hygroscopic loading protocol are shown here.

## 2. Materials and Methods

The experiments were conducted at the TOMCAT beamline of the Swiss Light Source (SLS), PSI Villigen in Switzerland. Samples were located inside an environmental chamber realized to fit the X-ray tomographic setup and described in [8]. Different sample preparation and experimental configurations to fully investigate the cell wall structure, the cell features and wood behaviour during hygroscopic loading in free or restrained swelling conditions are discussed in this work. To complete the information acquired by imaging, the actual MC in the wood cell wall at different environmental conditions was measured by vapour sorption analysis.

### 2.1. Samples Preparation

The wood under investigation is Norway spruce (*Picea abies* (L.) Karst). Thin toothpick-like pins wood samples were cut from cube samples of different dimensions depending on the scale of investigation: (1) cellular and (2) sub-cellular. Study (1) underlies the behaviour of cell wall structure during moisture changes of wood samples undergoing a free swelling during the hygroscopic loading protocol or subjected to a restraint to the swelling. Study (2) provides insights, for the first time, on the 3D structural changes of bordered pits during an entire loop in ad-/de-sorption. More details on sample preparation are discussed in this paragraph. Table 1 and Table 2 summarise the samples type and their dimensions.

*Study (1)* The investigation at the cellular scale was conducted under two conditions: (1.a) in free state and (1.b) with a restraint.

(1.a): The samples were prepared starting from a 10 × 10 × 10 mm^3^ wood cube with excellent alignment of the wood grain along the cutting directions, chosen to provide homogeneous specimens of earlywood and latewood. From this cube in wet state, 0.5 mm thick slices were cut with a microtome. A razor blade was then used to complete the cutting under a light microscope to produce thin toothpick-like pins of 500 μm × 500 μm × 8 mm in the tangential, radial and longitudinal directions, respectively. The individual wood cells were perfectly aligned along the longitudinal direction of the samples and each sample composed by cells of similar size. After cutting, the specimens were oven dried at 60 °C, vertically fixed to a sample holder using cyanoacrylate glue and then kept in a desiccator in a steady-state equilibrium with a salt-saturated aqueous solution, considered as the starting point for the experiments. Four samples of different densities were considered for this study: two denser latewood samples (LW1 and LW2) with thick cell walls and small lumens and two earlywood samples (EW1 and EW2) with thin cell walls and large lumens [10].

(1.b): Wood specimens were cut into sticks of 0.5 mm × 0.5 mm cross-section. The surface of the wood samples was as flat and smooth as possible, in order to fit in the slot (of 0.5 mm width and 2 mm length) of the restraining device (a cube of dimensions 4 × 4 × 4 mm^3^) and to be perfectly in contact with the cube slot’s surfaces. The samples were mounted on a translational stage and cut by razor blade in 0.5 mm slices and then into sticks. Finally, the samples were manually cut along 2 mm longitudinally to fit into the restraining device. Four wood samples, two of latewood (LWt and LWr) and one of earlywood (EWt), are located inside the polymethylmethacrylate (PMMA) cubes to receive a restraint to the free swelling in the tangential (t) or radial (r) directions. Initially, the wood stick samples were placed in a dry desiccator before their placement into the devices for testing. The PMMA cubes were used to restrain the free swelling of the wood samples undergoing relative humidity (RH) changes. (More details can be found in [17]).

**Figure 2:**
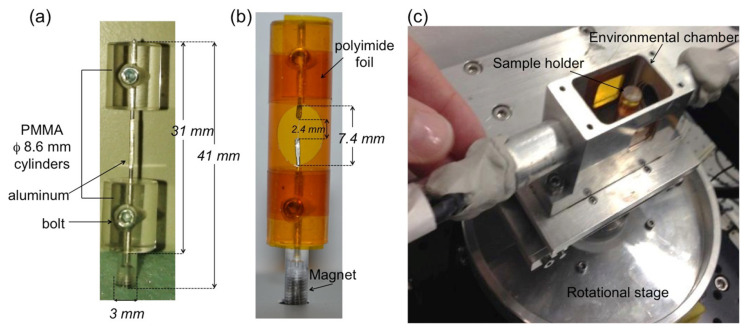
(**a**) Components of the sample holder, (**b**) finalized with polyimide foil and with access window for inserting and gluing the wood fiber. (**c**) A view from the top of the sample holder inserted in the environmental chamber.

*Study (2)* The wood thin sticks of 50 × 50 μm^2^ cross section were cut with a razor blade from 10 × 10 × 10 mm^3^ oven-dry cubes of the same wood specimen. The samples were exposed at hygroscopic loading protocol during the tomographic acquisition. Bordered pits were considered for anatomy characterisation in three earlywood stick samples: the first sample undergoing some steps in the hygroscopic loading protocol starting from its dried state–named sample A, while the second sample in green state (i.e., freshly cut from the tree and then frozen until the sample preparation)–named sample B; finally, a third sample taken in dried state and then subjected to a full hygroscopic loading protocol (i.e., from dried to wet in adsorption and then from wet to dried in desorption) during the experimental session–named sample C. Three pits in sample A are visible between ray cells, while one pit in sample B is found in the tracheid. The swelling/shrinkage behaviour of a bordered pit was analysed in sample C during a full sorption loop. As the thin wood sticks finely cut by razor blade tend to bend during moisture sorption, a holder was developed in order to keep the samples in place during the hygroscopic protocol. The wooden sticks were placed inside a cylinder-shaped sample holder of 8.6 mm diameter to allow a uniform X-ray path and remove the dependence on the rotational angle. The bottom part of the cylinder was equipped with a pin holder containing a magnet for insertion onto the magnetic rotational stage of the TOMCAT setup. The polyimide foil was rolled and glued around the two cylinders, kept together by a thin aluminum wire. A pre-cut circular hole allowed access inside the tube to host the wood sample, where the ends of the wood sample were glued to the pre-cut ends of the aluminum wire (Figure 2). The dried samples (e.g., samples A and C) were kept in a desiccator with desiccant particles until the beginning of the experiments.

The samples were exposed to RH cycles in adsorption and desorption during the tomographic scans.

### 2.2. Imaging Setups

The configurations used at the experimental setups of the synchrotron radiation-based phase-contrast X-ray tomographic micro- and nano-scope are schematized in Table 3 based on the case study listed in the previous paragraph. A description of the experimental setups used in cases 1.a and b is given in [10,17]. Details on the nanoscope setup for the case 2 are provided in this paragraph. In the three configurations, contrast is enhanced by in-line interference of the X-ray wavefront perturbed by the sample and detected as intensity pattern into the detector plane.

**Figure 3:**
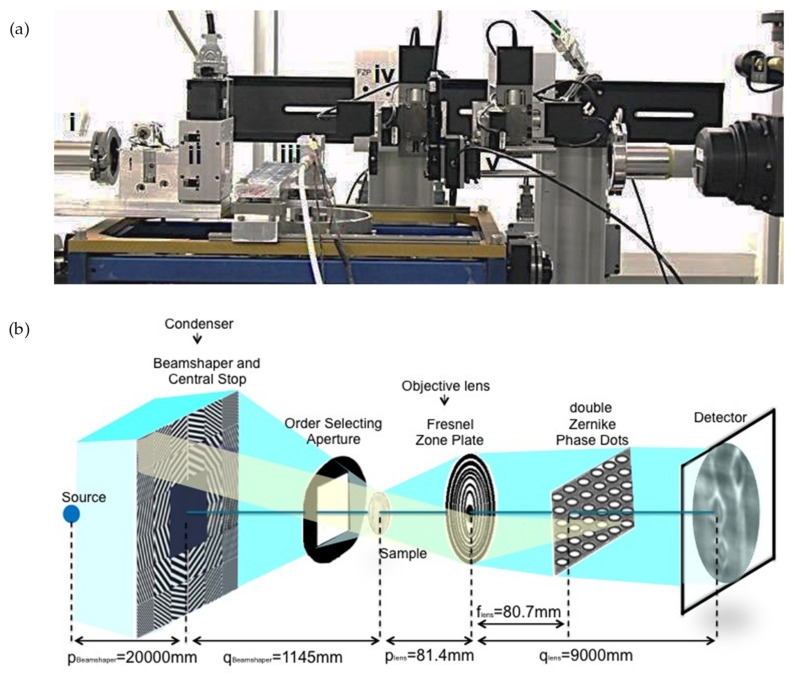
(**a**) Photo of the phase-contrast nanotomography setup at TOMCAT, showing (i) the end of the X-ray fly-tube, (ii) the condenser, (iii) the environmental chamber housing the wood sample, (iv) the Fresnel zone plate and (v) the Zernike phase dots. The camera is located at 9 m of distance from the (iv) and is not shown in the picture. (**b**) Schematic representation of the nanotomographic microscopy, with bordered pits imaged on the detector plane.

**Figure 4:**
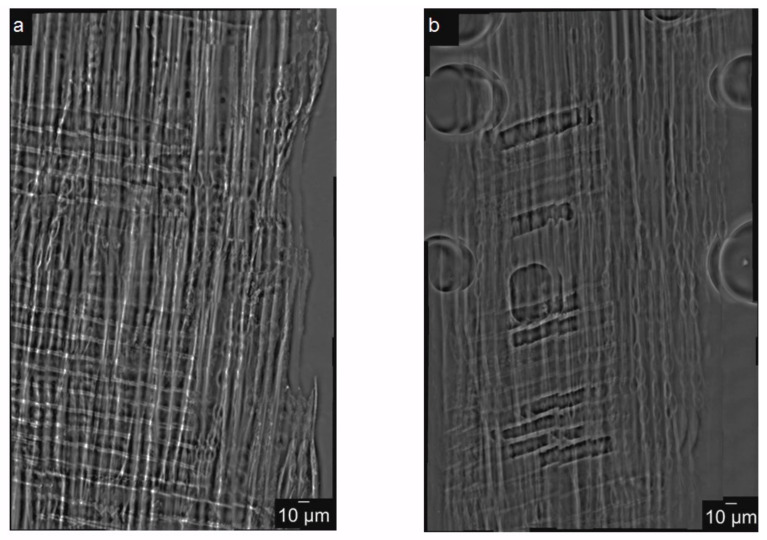
2D mosaic nanoimaging of the spruce wood. In (**a**) and (**b**) are depicted the 2D mosaic projections images, at 0° and 90° respectively.

The experimental protocol for imaging the bordered pits consists of two steps: (step 1) A preliminary region of interest (ROI) containing the pits in the wood cell wall was selected in each sample by using the PCO2000 detector with 20× magnifying lens to reach a pixel size of 0.38 μm. (step 2) A zoom into the selected ROI was made by removing the PCO2000 detector and using the nanoscopic configuration with 84 nm pixel size, as described in the next paragraph. Zernike phase-contrast hard X-ray nanotomography in the full-field mode [18] was applied to image the bordered pits in the cell wall of spruce wood. As shown in Figure 3, the key optical elements are a condenser, to provide a homogeneous and intense illumination at the sample position (focal point) and an objective lens, to magnify the image of the sample on the detector. The optimum contrast/resolution is achieved by matching the numerical aperture of the illumination with that of the objective lens. Schematically, a beam shaper of dimension 1.5 × 1.5 mm^2^ collimates the beam to approximately 30 × 30 μm^2^ top-flat square illumination on the sample. The Fresnel zone plate (FZP) of 100 um diameter and 70 nm outmost zone width magnifies this region into the detector plane located at 9 m further downstream. Phase enhancement in the full-field microscope is achieved by letting the beam passing through Zernike Phase Dots positioned in the back-focal plane of the objective FZP. They consist of a dot array aligned to the undiffracted fraction of the beam in the Fourier space (=back-focal plane of the lens), The structure height of the dots leads to a phase shift of π/2 at 12 keV. The sample is located at the focal distance of the condenser which corresponds as well at the focal distance of the FZP. A Photonic Science VHR Image Star X-ray camera based on a full-frame transfer Kodak charge coupled device (CCD) with 3056 × 3056 pixels of 12 × 12 μm^2^ size is located downstream and collects the raw projections with a 14 bit dynamic range. The camera features a full well capacity larger than 110 ke-/pixel with a readout noise at 8 MHz of less than ten electrons and dark current smaller than 0.5 electrons/pixel/second. A film of 2.5 mg/cm^2^ GdOS:Tb is deposited on a fiber optic taper (FOP), resulting in a final assembly of 12 × 12 mm^2^ sized active input window with a nominal pixel size of 4 μm. Taking into account the magnification M = 110× of the microscope, the field-of-view (FOV) was 12 × 12 μm^2^ with an effective pixel size of 36 × 36 nm^2^. For faster acquisition, the 3× binning factor was employed, resulting in a final effective pixel size of 84 nm which allowed to capture the main features of the bordered pits (torus and pit openings). The detector acquired 541 (plus 5 dark fields and 20 flat fields) radiographic projections at equiangular positions over a total rotation angle range of 180° with an exposure time of 800 ms. The reconstruction was performed using the GridRec algorithm [19]. To complete the series of data, a 2D ‘mosaic nano-imaging’ has been performed on two datasets. This technique is well-suited to reconstruct samples with cross-sectional sizes bigger than the FOV. It consists of stitching projections of the same sample taken at two different angles, 0° and 90° around the vertical axis, and in different regions to cover the entire sample area. The exposure time was halved to reduce possible effects of dose deposition on the sample during multiple scans. Figure 4 depicts an example of 2D mosaic image resulting from raster scanning the sample in full-field microscopy mode. It results from collecting a total of 10 × 16 = 160 radiographic images covering the entire area of a spruce wood sample with lateral extension of 266 μm ca. The procedure can be applied at any angular positions, as described in [20]. In this case, an area of 292.32 × 467.71 μm^2^ was collected at the two orthogonal angles. The stitching procedure was then performed through the Fiji plugin ‘Stitch Grid’.

### 2.3. Dynamic Vapour Sorption Analysis

The dynamic vapour sorption (DVS) analysis was performed with the VTI-SA+ Vapour Sorption Analyser in order to record the sample mass at each RH value, thus obtaining the actual MC. Such analysis was done after each beam tests in which the samples were exposed to a full hygroscopic loop in adsorption and desorption down to its dried state and this state was maintained by inserting the samples in a desiccator with desiccant particles (RH = 0%) until the start of the DVS measurements. As the bottom part of each wood sample was glued to the TOMCAT sample stage during the beam test, DVS analysis was performed only to the top portion of the samples (to exclude the influence of cyanoacrylate glue to such analysis). Finally, the wood samples were exposed to the same RH steps of the full sorption loop to which they were subjected during the beam tests in order to compare both analyses. The results obtained were used for calculate the swelling coefficients derived from linear fits on strains versus MC results.

### 2.4. Methods of Analysis

At the cellular scale, the analysis includes: (A) porosity measurements and (B) deformations calculated at the global and local levels.

(A). The wood porosity, due to the presence of the lumen, was defined here as void volume per total volume. By grey value thresholding, the tomographic datasets were first converted into binary datasets composed of voxels containing either cell wall material or air. The “region growing” algorithm available in VGStudio MAX 2.0 software was used as a thresholding method. Starting from the binary images, the wood volume was calculated by counting the number of material (white) voxels for each slice, summing up over all the stacked slices, and multiplying this value by the voxel size.
(B). The wood cell wall deformations were calculated both at the global level (such as during free swelling/shrinkage) and at the local level (as observed during the restraining experiment). In particular, for case 1.b, two ROIs were selected for each sample containing different restraint intensity to the free swelling from the restraining device. In detail, regions of the sample not directly in contact with the PMMA cube surface received a minor restraint to the swelling during moisture adsorption caused by the device; such regions are called “ROIs 1”. Conversely, regions directly in touch with the PMMA cube surface were more subjected to the restraining role of the device: such regions are referred to as “ROIs 2”. The most commonly used transformation method for the calculation of the global deformations is the so-called affine transformation, where changes in position, size and shape of a volume are described. The parameterisation of a 3D affine transformation generally involves 12 parameters (three for defining translation, three for rotation, three for scaling and three for shear). Details are published in [8,10]. A previous work of the author [21] describes the quantitative method implemented to analyse the local deformations in cellular wood tissues when subjected to environmental changes. The algorithm, written in Matlab, employs a free-form deformation model based on B-splines. This algorithm was then used to detect the local strains in bordered pits as well.

## 3. Results

### 3.1. Wood Porosity

The porosity was calculated in the wood 3D datasets at the reference state (e.g., lowest RH level in adsorption) as described in paragraph 2.4. Results are reported in Table 4 for wood datasets acquired in the two cases under investigation, e.g., free swelling (1.a) and restrained swelling (1.b), by using the microscope setup configuration of the TOMCAT beamline. The sample porosity varies between 45% in latewood up to 78% in the earlywood tissue.

### 3.2. Anisotropic Swelling Behaviour of Earlywood and Latewood

**Figure 5:**
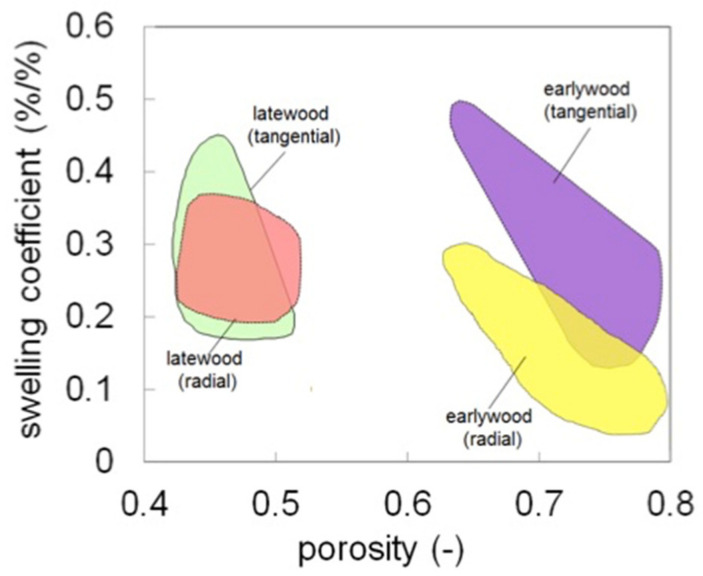
Swelling coefficients of latewood and earlywood for different porosity. The plot collects the results discussed in Section 3.2 and in previous works taken from different sources on softwood species [23,24,25].

The main findings regarding the anisotropic behaviour of different wood tissues are summarised in this paragraph. The anisotropic swelling ratios, i.e., the ratio between the tangential to the radial swelling, discussed in previous works [8,10] are reported in Table 5 for the samples investigated at the cellular scale. Earlywood swells anisotropically during moisture sorption. Conversely, latewood shows quite isotropic swelling behaviour. The anisotropy of earlywood and latewood tissues is importantly modified in restrained swelling experiments due to the reduction of swelling percentage along the restraining direction. The linear swelling coefficients for dry wood, defined as the slope of the linear relation between strains and MC, are plotted in Figure 5. The swelling coefficient ranges from 0.1 to 0.5, with lower values for earlywood in radial direction, which is attributed to the restraining role of the ray cells. There is no direct relationship between the tangential swelling coefficient and the porosity, whereas a slight decrease of the radial swelling coefficient with the sample porosity has been observed. This result indicates that the cellular structure also plays an important role. Such cellular structure features must include anisotropy of cell layer materials, layered structure of the cell wall and geometry of the cell.

**Figure 6:**
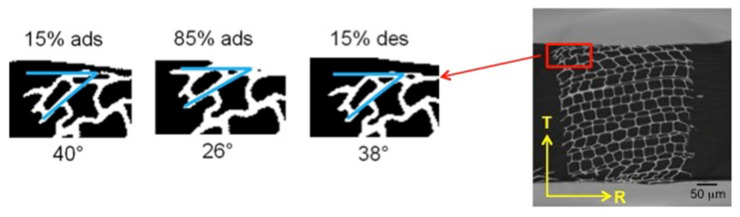
Shape-memory effect on the cell undergoing large deformations in a ROI of EWT. The ROI was selected out of 2D reconstructed images and binarized.

**Figure 7:**
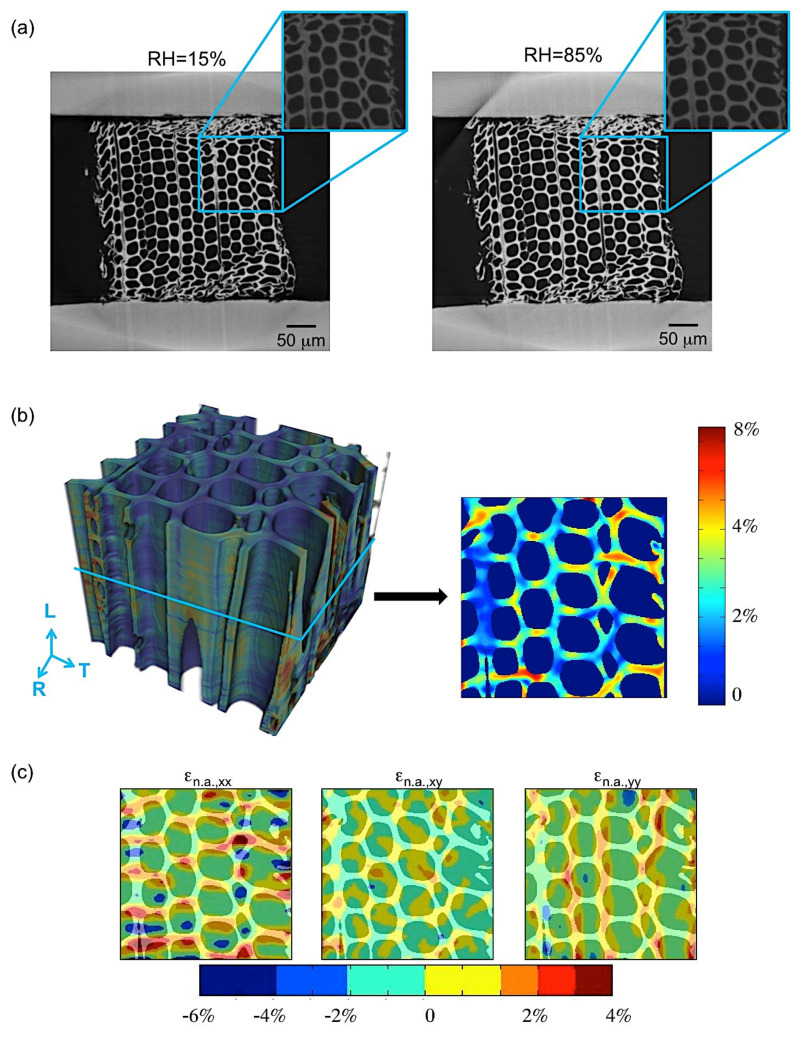
(**a**) LWR sample, dry (left) vs. wet (right), with a zoom on the ROI (in blue) where shape-memory is observed, (**b**) 3D map of the equivalent non-rigid strains, with a slice cut out in the middle of the volume and (**c**) the components of the non-rigid strains in tangential (xx) and radial (yy) directions.

Two important phenomena, generally occurring in many biological materials, were observed during restrained swelling experiments, i.e., the buckling effect [17] and the shape-memory. Results of shape memory are discussed in this section. Shape memory was observed in a group of cells subjected to wetting and drying (Figure 6). The angle formed between the wood cells and the plane parallel to the device slot is equal to 40° at 15% RH, 26° at 85% RH in adsorption and goes back to 38° at 15% RH in desorption. This means that, during re-wetting, the cells subjected initially to a large deformation caused by razor blade cutting during the sample preparation, then try to regain their original cell shapes (also known as shape memory) leading to high local non-rigid deformation. The analysis of this phenomenon has been performed on a specific ROI of the LWr sample of case 1.b adjacent to the restraining device (Figure 7). The quantification of the local deformations is also reported in Figure 7b,c. Figure 7b shows the 3D map of the equivalent non-rigid strains and a slice cut out in the middle of the volume. The equivalent strain is a qualitative analysis to describe the deformation intensity in wood [22]. The components of the non-rigid strains are mapped on the same slice in Figure 7c. In Figure 7b (right), high equivalent non-rigid strains in the cell walls at the outer edge are observed. A comparison with Figure 7c shows that these strains are due to high tensile non-rigid strains, n.a.,xx in horizontal cell walls and, to a less extent, to high tensile strains, n.a.,yy, in vertical cell walls. These tensile strains cannot be explained by the restraining of the PMMA device, but only by a deformation induced by shape memory. We consider a possible realignment of the different polymers [9] to justify the phenomena.

### 3.3. Anatomy of Bordered Pits in Earlywood Samples

The visualisation of bordered pits in earlywood samples was quite trivial [26]. The two-steps procedure explained in Section 2.2 was carefully performed in order to highlight the occurrence of pits in the wood cell wall. After acquisition with the nanoscope setup, the reconstructed tomographic data were resliced along the radial-longitudinal plane for visualizing the structure of the bordered pits. Four pits were visualised in two earlywood samples: one sample (here named sample A) underwent some steps in ad-/de-sorption (10–50% in adsorption and 50–25% in desorption), while the second sample was scanned in its green state (sample B).

**Figure 8:**
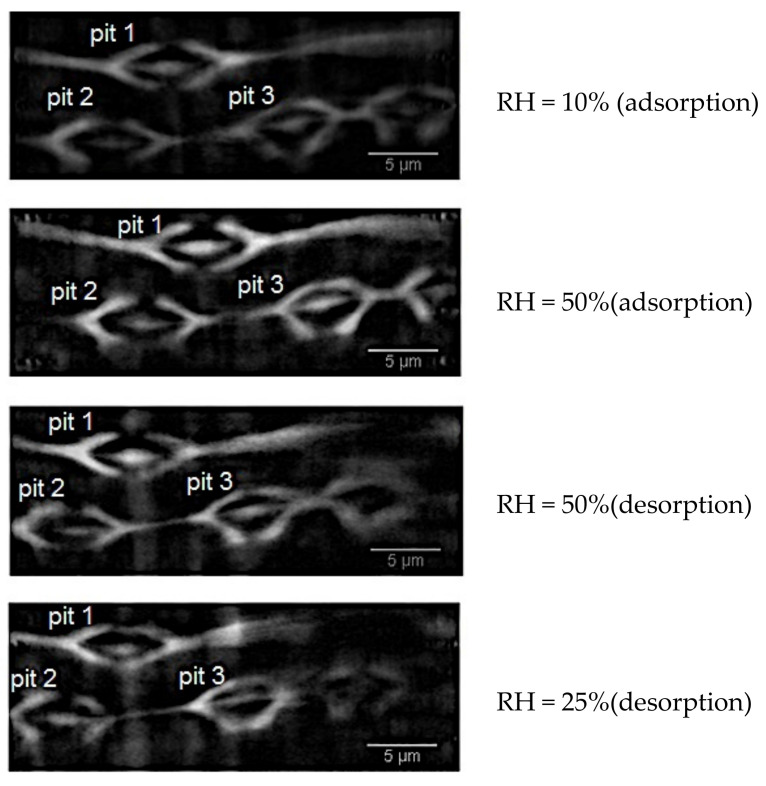
Cross-sectional slice of Sample A at different RH states. In the figure, the three pits analysed in the paper are indicated.

A set of 15 slices for each dataset of sample A was analysed in 2D with the Fiji software. For this set of data in which three pits were visible, dimensional changes of the torus size, pit aspiration and pit opening were calculated in adsorption (RH = 10% and 50%) and in desorption (RH = 50% and 25%), Figure 8. With a similar approach, the green wood pit of sample B was analysed. In sample A, the torus seals one of pit aperture and pit 1 moves towards pits 2 and 3. As a consequence, the water vapour flows in the space between pit 1 and pits 2–3. The pits result to be aspirated in dried state, while unaspirated in wet state; the torus is positioned perfectly in the pit centre at RH = 50%. In this position, water vapour can pass through the margin from one cell to the other one and the pit gets open. During drying, the pit aspirates. By surface tension, the pit membrane opens to the same extent of its closing during the aspiration process.

**Figure 9:**
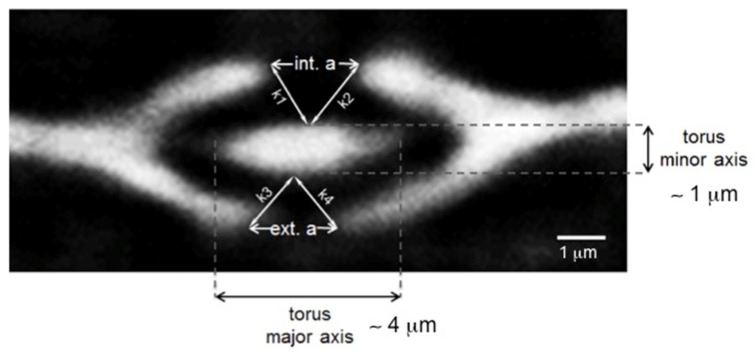
Re-slice of the sample A tomographic dataset, with a ROI cutting in the position of pit 1. Bordered pit 1 in section transverse to the pit membrane, showing the k parameters reported in Table 6.

The torus is described as an ellipse in the transverse plane (see pit 1 in Figure 9). The three pits of sample A show the following tendency during a full hygroscopic loop (RH = 10–50% in adsorption and 50–25% in desorption): a decrease of the major axis and an increase of the minor axis. The internal aperture (int. a) indicates the region of the pit aperture where the aspiration process occurs, whereas the opposite side is named external aperture (ext. a). In pit 1, int. a decreases in adsorption in order to prevent the fluid transport in the pit chamber while it increases in desorption. Conversely, ext. a increases both in adsorption and in desorption. In pit 2, int. a behaves similarly to pit 1, while ext. a gets smaller. At higher RH, the torus falls on one side of the pit aperture, with a consequence closure of the aperture on one side and an opening on the opposite side due to a pressure effect. The pit aspiration process of pit 1 is quantified by calculating the distances k1, k2, k3 and k4 indicated in Figure 9. These parameters are reported in Table 6. While k1 and k2 increase in adsorption and in desorption, k3 and k4 tend to decrease, thus demonstrating that the aspiration process occurs at the bottom of the pit aperture. This confirms that the water vapour flows between Pit 1 and Pits 2 and 3.

**Figure 10:**
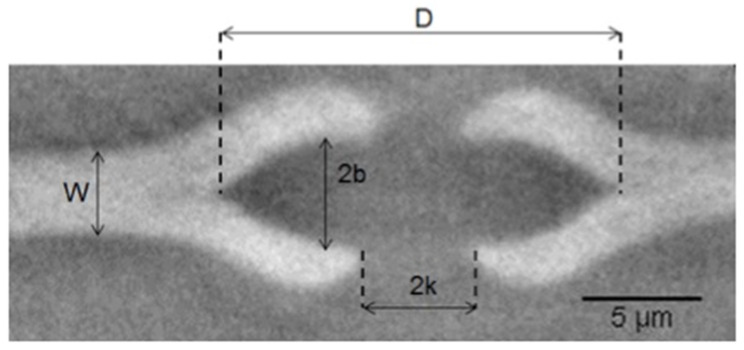
Bordered pit in sample B in section transverse to the pit membrane. W is the pit membrane thickness, D the major axis of the ellipse, 2b the minor axis and 2k the pit aperture. Torus was not probed in this case.

In sample B, only one pit was visualised between the tracheid cells of the earlywood sample B. Figure 10 shows the dimensions calculated for a set of 15 slices and reported in Table 7.

### 3.4. Behaviour of Bordered Pits during Moisture Sorption

**Figure 11:**
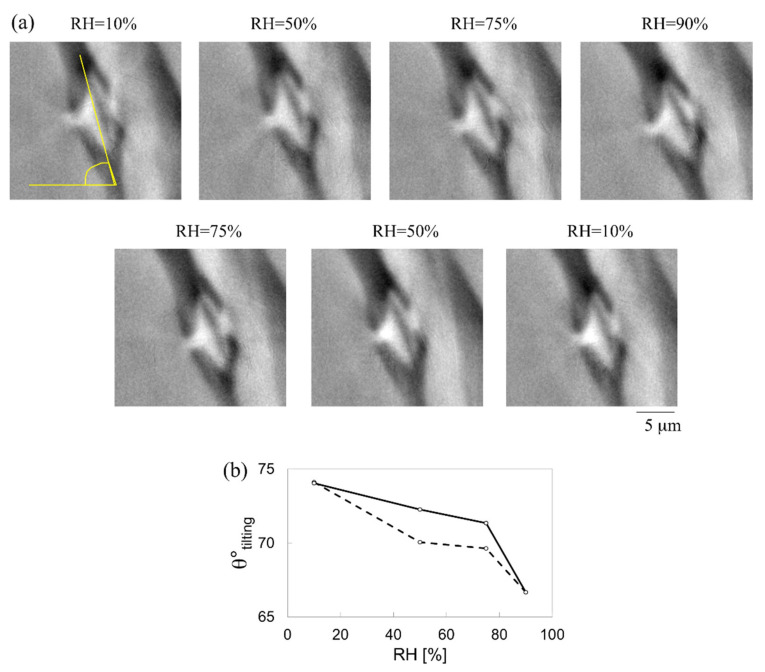
(**a**) Cross-sectional view of pit 1 at different RH values in adsorption (10–50–75–90%) and in desorption (75–50–10%) used for the analysis of (**b**) the tilting angle in adsorption (dashed line) and in desorption (continued line).

The behaviour of a bordered pit in an earlywood sample (sample C) was studied during a full hygroscopic loading protocol. The pit presents a diameter of 9 μm at 10% RH. The non-rigid strains are calculated for the entire loop: 10–50–75–90–75–50–10%, with RH = 10% in adsorption considered as the reference state. Figure 11a shows a cross-section of the bordered pit at different RH levels. The pit is slightly tilted (θ° tilting) with respect to the horizontal axis (as indicated in Figure 11a,b) with an angle of 74° at 10% RH. This angle decreases to 67° at 90% RH during adsorption, and returns to its original value at 10% RH in desorption, as shown in Figure 11b.

Further studies on the deformation of bordered pits during hygroscopic cycling and its effect on permeability can bring new insights on the change of permeability in dried wood caused by subsequent wetting/drying.

## 4. Discussion

The multi-scale approach. This novel method combines the technologies available at the TOMCAT beamline, which provides insights on wood anatomy and hygro-mechanical behaviour at micro- and nano-scales with an image analysis approach. The methodology of analysis consists of quantifying the affine and non-rigid deformations occurring at the cell wall level. The morphology of the bordered pits was investigated on the earlywood sample, thus providing insights on the aspiration process.

The wood behaviour. At the cellular scale, the cell wall material in dried state has a low porosity where water molecules can find readily available sorption sites. For further sorption to take place, i.e., above 2–3% moisture content, the sorption of water molecules pushes the polymeric components apart (phenomena not directly visible at such investigation scales), resulting in an increasing porosity, assumed to be totally filled by water (saturated porosity). Anisotropy strongly depends on the porosity. Earlywood samples present the highest porosity and very thin cell walls, manifesting the highest swelling anisotropy. The strains are strongly dependent on the swelling properties of wood at the different moisture content values. This is further confirmed in the restrained swelling experiments (case 1.b). The restraining device successfully prevents the free swelling of wood during moisture adsorption, thus significantly modifying the anisotropy of swelling. Two interesting phenomena are observed: the buckling effect and the shape memory. In (case 1.b), the wood cells at the edges of the sample and in contact with the restraining surface undergo buckling during water sorption. Buckling occurs both in latewood and in earlywood tissues, but to a lesser extent in the bulky latewood. Such deformation was related, most likely, to an initial disturbed state due to the sample cutting and insertion into the restraining device. Such a state must facilitate the occurrence of buckling. A buckling mode of axial compressive type is found in a group of cells, which are subjected to restraint along the radial direction. More detail about the buckling effects are discussed in [17,23], reporting high values of non-affine strain (~8%) where buckling occurs. The observed buckling accords well with the typical buckling mode of regular periodic honeycomb structure under radial restrain as reported in [17,18,19,20,21]. Indeed, this buckling mode is characteristic for honeycomb structures subjected to axial compressive stresses. Shape-memory effect is observed in initial highly deformed cells due to sample preparation, which regain their original cell shape when rewetting. This memory effect is probably related to a realignment of polymers. It may be that during sample preparation, the cell wall was deformed, resulting in a warping of the stiff cellulose fibrils. Fibrils are less sensitive to moisture compared to the matrix, due to their high content in crystalline hydrophobic cellulose. Thus, it is suspected that fibrils can remember their original relative position and geometry. A “permanency” of the geometry could be embedded in the cellulose fibrils at the time of its synthesis by the living cells and its deposition in the external skeleton [25]. Such a hypothesis was also considered in other work [26]. The shape memory effect has also been observed in the bordered pit during a full hygroscopic loop, as visualized in Figure 11a. This effect explains that the wood sample housing the bordered pit returns to its initial position after being subjected to an adsorption and desorption cycle. Further studies on the deformation of bordered pits during hygroscopic cycling and its effect on permeability can bring new insights on the change of permeability in dried wood caused by subsequent wetting/drying.

## Figures and Tables

**Table 1:** A summary of the samples used for the investigation at micro-scale of the wood swelling in free state (1.a) and under restraint (1.b) with their respective dimensions along the tangential (T), the radial (R) and the longitudinal (L) directions.

Study (1) Cases:	Earlywood (EW)	Latewood (LW)	Dimension [T × R × L]
**(1.a)**	EW1, EW2	LW1, LW2	500 μm × 500 μm × 8 mm
**(1.b)**	EWt	LWt, LWr	500 μm × 500 μm × 2 mm

**Table 2:** A summary of the samples used for the investigation at nano-scale of the bordered pits morphology (samples A and B) and swelling/shrinkage behaviour (sample C) with their respective dimensions along the tangential (T), the radial (R) and the longitudinal (L) directions.

Sample Name	Sample A	Sample B	Sample C
**pits no.**	3	1	1
**initial state**	dried	green	dried
**dimensions**	50 μm × 50 μm × 3 mm	50 μm × 50 μm × 3 mm	50 μm × 50 μm × 3 mm

**Table 3:** SDD is the sample to detector distance, which plays a role for contrast enhancement. * In case 2, a selection of a region of 470 × 392 pixels size over the total camera field of view (FoV) was made, matching the illuminated region of interest.

Study	Cellular Scale		Sub-Cellular Scale
	Case 1.a	Case 1.b	Case 2
Detector	PCO 2000	PCO 2000	Photonic Science VHR Image Star
**FOV [μm^2^ × μm^2^]**	757 × 757	757 × 757	12 × 12 *
**Binning**	1	2	1
**Effective Pixel size [μm]**	0.37	0.74	0.084
**Exposure time [ms]**	75	40	1200
**SDD [mm]**	30	20	see Figure 3
**Projections nr.**	1001	1001	541
**X-ray Energy [keV]**	20	20	12

**Table 4:** Earlywood and latewood porosity in case of free swelling (1.a) and restrained swelling (1.b)**.**

Case (Cellular Scale)	Sample Name	Porosity [%]
Case 1.a)	EW1	78
	EW2	64
	LW1	50
	LW2	45
Case 1.b)	EW_T_	65
	LW_T_	54
	LW_R_	60

**Table 5:** Anisotropy of earlywood and latewood in free swelling and of regions of wood with minor (ROI 1) or major (ROI 2) restraint.

	Case 1.a)		Case 1.b)	
Anisotropy	Free Swelling		Restrained Swelling	
	1	2	ROI 1	ROI 2
EW	3.10	1.70	EW_T_:2.40	EW_T_:0.25
LW	1.30	1.10	LW_T_:0.80 LW_R_:5.30	LW_T_:0.50

**Table 6:** Distances between torus and pit membrane (k parameters) at different RH with their respective standard errors. The numbers are calculated for pit 1 in Figure 8 and Figure 9.

State	RH = 10% (ads.)	RH = 50% (ads.)	RH = 50% (des.)	RH = 25% (des.)
k_1_ ± σ [μm]	1.00 ± 0.02	1.20 ± 0.04	1.40 ± 0.03	1.42 ± 0.04
k_2_ ± σ [μm]	1.22 ± 0.05	1.36 ± 0.05	1.38 ± 0.06	1.60 ± 0.08
k_3_ ± σ [μm]	1.23 ± 0.18	1.08 ± 0.04	1.05 ± 0.05	1.00 ± 0.04
k_4_ ± σ [μm]	1.54 ± 0.05	1.20 ± 0.05	1.03 ± 0.05	0.90 ± 0.05

**Table 7:** W, D, 2b and 2k in sample B. Mean values with their corresponding standard deviations.

Measurements	W	D	2b	2k
**Value ± 0.05 [μm]**	3.40	16.40	4.25	4.50

## Data Availability

Data are available under request.
